# Congenital Syphilis Like Many Years Ago

**DOI:** 10.1155/2011/235059

**Published:** 2011-09-22

**Authors:** Giulia Brighi, Giorgia Farneti, Antonella Marangoni, Elisabetta Tridapalli, Iria Neri, Maria Grazia Capretti, Giacomo Faldella

**Affiliations:** ^1^Neonatal Intensive Care Unit, S. Orsola-Malpighi Polyclinc, University of Bologna, 40138 Bologna, Italy; ^2^Microbiology Section, DESOS, S. Orsola-Malpighi Polyclinc, University of Bologna, 40138 Bologna, Italy; ^3^Division of Dermatology, Department of Internal Medicine, Geriatrics and Nephrology, University of Bologna, 40138 Bologna, Italy

## Abstract

This case concerns a premature infant with typical signs of congenital syphilis born to an untreated foreign mother. Syphilis prevalence in pregnant women has been rising in Italy since the beginning of the 21st century, mainly due to immigration. A correct antenatal syphilis screening and consequent adequate therapy of pregnant woman are fundamental to prevent the neonatal infection.

## 1. Introduction

Syphilis among pregnant women, and the consequent congenital syphilis (CS), are now reemerging in Italy, due to recent migration dynamics from Eastern Europe [1]. Congenital syphilis (CS) is mainly a consequence of the lack of antenatal care and control of sexually transmitted infections. The bedrock of the prevention of CS is the performance of syphilis serological screening during pregnancy, making appropriate treatment possible and preventing vertical transmission [2]. According to the Italian legislation (Decreto Ministeriale 10/09/1998) [3] all the pregnant women should be tested in the first trimester. Several factors (e.g., maternal infection stage, gestational age, and maternal treatment) contribute to the different manifestations of congenital syphilis. Infected infants can be asymptomatic or can show subtle and insidious findings or multiple-organ involvement. Even asymptomatic newborns can develop early or late postnatal manifestations [4]. Few cases, like the one described here, present the typical features of symptomatic congenital syphilis with cutaneous manifestations, bone lesions, and prematurity.

## 2. Case Presentation

In January 2009 a woman from Romania, in her 31st week of gestational age, was admitted to the obstetric department of our hospital for preterm delivery. She gave birth, by cesarean section, to a female weighing 1881 g, with an Apgar score of 7 after 1 min and 9 after 5 mins. 

**Figure 1:**
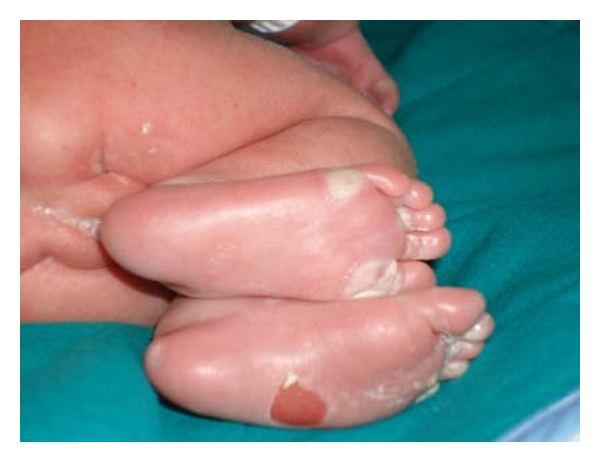
Typical blistering skin lesions on the soles of feet in the case described.

At birth, on physical examination cutaneous lesions were evident, consisting of a maculopapular rash and blisters on the arms and legs with superficial desquamation particularly on the palms and soles (Figure 1).

The baby was immediately transferred to our Neonatal Intensive Care Unit (NICU). At the admission in NICU she was given nCPAP because of respiratory distress that was continued for 24 hours. A lumbar puncture was performed; the CSF was characterized by 18 leukocytes, 84 mg/dL protein, and 36 mg/dL glucose; CSF TPHA (1/80 titre) and *Treponema pallidum *IgG western blot (WB) were both positive, as well as VDRL and *T. pallidum PolA* PCR. Haematochemical investigations showed an increase in leucocyte number (27.440/microl, *N* 36.1%, *L* 42.1%) and C reactive protein (CRP) levels (16 mg/dL, normal value < 0.8 mg/dL) with normal haemoglobin, red blood cell, and platelet count; tests of liver and kidney function were also normal. The newborn syphilis serology showed positive RPR (1/32 titre), *T. pallidum *IgM WB [5], and TPHA (titre > 1/640). 

**Figure 2:**
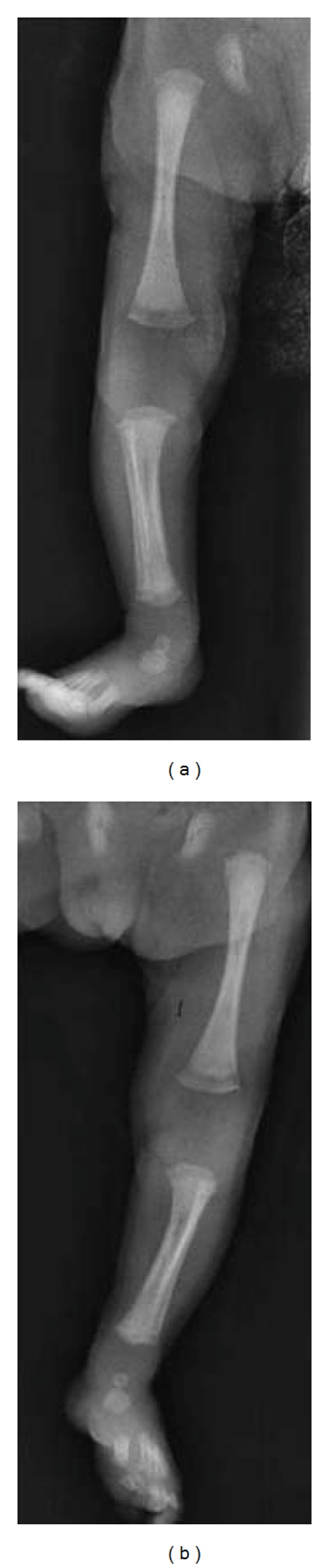
X-ray examination of the long bones of the case described.

The limited extension of the left knee was suggestive for long-bone lesions; indeed, the radiographic examination showed signs of osteochondritis and periostitis at metaphyseal level (Figure 2). Cerebral US, EEG, heart US, and eye examination were normal.

The maternal history revealed that she received no antenatal care and she had been investigated for syphilis only few days before (TPHA > 1/640, reactive RPR test, with a titre of 1/16).

Despite positive serology, there was no time for adequate treatment because she had preterm unstoppable labour. 

The newborn was treated with i.v. aqueous crystalline penicillin G for 14 days, Amikacine for 10 days, and IgM-enriched immunoglobulins for 3 days.

After discharge the baby was enrolled in a clinical and serological followup.

Brain MR, performed at 2 months of life, was normal; psychomotor development at 2 and 8 months of life was adequate for the age. The lumbar puncture performed at 6 months of life showed negative treponemal and non-treponemal tests on CSF. At her last examination (18 months of life) RPR turned negative while *T. pallidum* IgG WB and TPHA (1/320 titre) were still positive; she had a normal growth (length 50th pc, weight 50th pc).

In Table 1 the baby's characteristics at birth and follow-up visits are summarized. 

## 3. Discussion

This paper presents the typical features of symptomatic congenital syphilis with cutaneous manifestations, bone lesions, and prematurity. Diagnosis of CS is often difficult because children are usually asymptomatic at birth and prematurity may be the only clinical manifestation. In our previous papers [1, 6], we reported 6 cases of congenital infection; two out of these six infants had a positive VDRL test in CSF, another one presented long bone lesions at X-ray examination, whereas the remaining 3 newborns were preterm (GA: 26 weeks, 28 weeks, and 31 weeks). All newborns had positive IgM at WB assay. Since the beginning of 21th century, the present case has been the only one in our hospital with cutaneous lesions evident at birth, reminding us of images seen in old infectious diseases textbooks. Recognition of CS can be difficult, first, as a result of lacking experience in clinicians and, second, due to the nature of the disease as the “great imitator,” therefore often presenting with nonspecific clinical signs and symptoms. In a recent paper of Tridapalli et al. [7] data from a multicenter Italian study have been reported in order to evaluate the incidence of congenital syphilis. Eight out of the twenty-five infected babies presented clinical signs at birth, but very few presented typical skin lesions. 

The case described in the present paper occurred in a woman from Eastern Europe, confirming that in our country syphilis serum prevalence in pregnant women is strongly related to migration flows from endemic countries [1, 6, 7]. We can affirm that a correct antenatal syphilis screening is of primary importance to identify serum-positive women and to assure adequate treatment to prevent the risk of vertical transmission [6–8]. 

## Figures and Tables

**Table 1:** Infant's characteristics at birth and follow-up visits.

	Birth	1 month	2 months	3 months	5 months	6 months	8 months	12 months	18 months
Syphilis serology									
TPHA	> 1/640	1/640	1/640	1/320	1/320	1/320	1/320	1/320	1/320
RPR	1/32		1/4	1/2	1/1	Negative	Negative	Negative	Negative
WB IgG	Positive	Positive	Positive	Positive	Positive	Positive	Positive	Positive	Positive
WB IgM	Positive	Positive	Positive	Negative	Negative	Negative	Negative	Negative	Negative
Lumbar puncture									
VDRL	1/80					Negative			
TPHA	Positive					Negative			
WB IgG	Positive					Negative			
WB IgM	Positive					Negative			
Radiographic examination	Signs of osteochondritis and periostitis at metaphyseal level								
Brain MR			Normal						
Cerebral US	Adequate for gestational age	Normal		Normal	Normal	Normal			
EEG	Normal		Normal						
Heart US	Normal								
Eye examination	normal	Normal		Normal					
Apgar score	7 after 1 min; 9 after 5 mins								
Weight	1881 g	2240 g	3680 g	4500 g	5900 g	6200 g	7900 g	9500 g	10800 g
Length	42 cm	46.5 cm	51 cm	55 cm	59 cm	61 cm	65 cm	71 cm	79 cm

## Abbreviations

Brain MR: Brain magnetic resonance
Cerebral EEG: Electroencephalogram
Cerebral US: Cerebral ultrasonography
CRP: C reactive protein
CS: Congenital syphilis
CSF: Cerebral spinal fluid
nCPAP: Nasal continuous positive airway pressure
Heart US: Heart ultrasonography
IgG: Immunoglobulin G
IgM: Immunoglobulin M
i.v: Intravenous
NICU: Neonatal intensive care unit
PCR: Polimerase chain reaction
RPR: Rapid plasma reagin
TPHA: Treponema pallidum haemoagglutination assay
VDRL: Veneral disease research laboratory
WB: Western blot.
